# Current status of sublingual immunotherapy in the United States

**DOI:** 10.1186/1939-4551-7-24

**Published:** 2014-10-08

**Authors:** Shelby Elenburg, Michael S Blaiss

**Affiliations:** Departments of Pediatrics and Internal Medicine, Division of Clinical Immunology and Allergy, University of Tennessee Health Science Center, 7205 Wolf River Blvd, Germantown, TN 38138 USA

**Keywords:** Sublingual immunotherapy, Immunotherapy, Allergic rhinitis, Allergic rhinoconjunctivitis, Grass, Ragweed

## Abstract

Sublingual immunotherapy (SLIT) use in the United States to date has been limited, despite common use and demonstrated efficacy elsewhere in the world. This is largely in part due to lack of FDA-approved SLIT products, lack of established dosing and administration guidelines, and cost concerns. Several recent studies have demonstrated efficacy and safety of two sublingual grass tablets and one ragweed tablet approved by the FDA, and one sublingual ragweed liquid currently pending FDA approval. With FDA approved SLIT products, there will be numerous challenges to the allergist and patient in deciding whether to pursue SLIT or SCIT (subcutaneous immunotherapy) for allergic rhinitis. This review highlights the current state of SLIT in the United States, and expected future directions.

**Electronic supplementary material:**

The online version of this article (doi:10.1186/1939-4551-7-24) contains supplementary material, which is available to authorized users.

## Introduction

Sublingual immunotherapy (SLIT) is commonly used in many parts of the world, but use in the United States to date has been limited. There have been numerous SLIT studies performed to date, but, until recently, only a small number have been double-blind, placebo-controlled. This review sought to focus on the current state of SLIT in the United States, and expected future of SLIT pending Food and Drug Administration (FDA) approval of sublingual therapies.

### Current immunotherapy use in the United States

In 2011, a survey of practicing allergists in the American College of Allergy, Asthma, and Immunology (ACAAI) [1] showed that 11.4% of respondents were commonly using SLIT, compared to 5.9% in a 2007 survey [2]. 90.2% of respondents in the 2011 survey found lack of FDA approval for SLIT products to be the primary barrier to SLIT use in the United States. Other barriers included lack of established practice parameters, unknown effective dose, inadequate training or experience, cost, dose required, and adverse effects. SLIT was felt to be safer than subcutaneous immunotherapy (SCIT) by 66.7% of respondents. As there have not been FDA-approved vehicles for administration of SLIT, practitioners using SLIT have had to use other commercially available products, primarily extracts available for SCIT (86%). A majority (76.5%) of those using SLIT used multiple allergen extracts in all or at least half of their patients, versus only 23.5% using monotherapy in all or at least half of patients. 80% of physicians using SLIT required at least 1 dose to be administered in the office, and of those, 67% required only the first dose to be given in the office. Only 13.3% required 3 or more doses to be done in an office setting before home use. In terms of effectiveness, no physicians felt it was not effective at all. 56.9% felt SLIT was less effective that SCIT, though 31.4% thought the two were equally effective. 11.8% felt SLIT was more effective.

**Figure 1:**
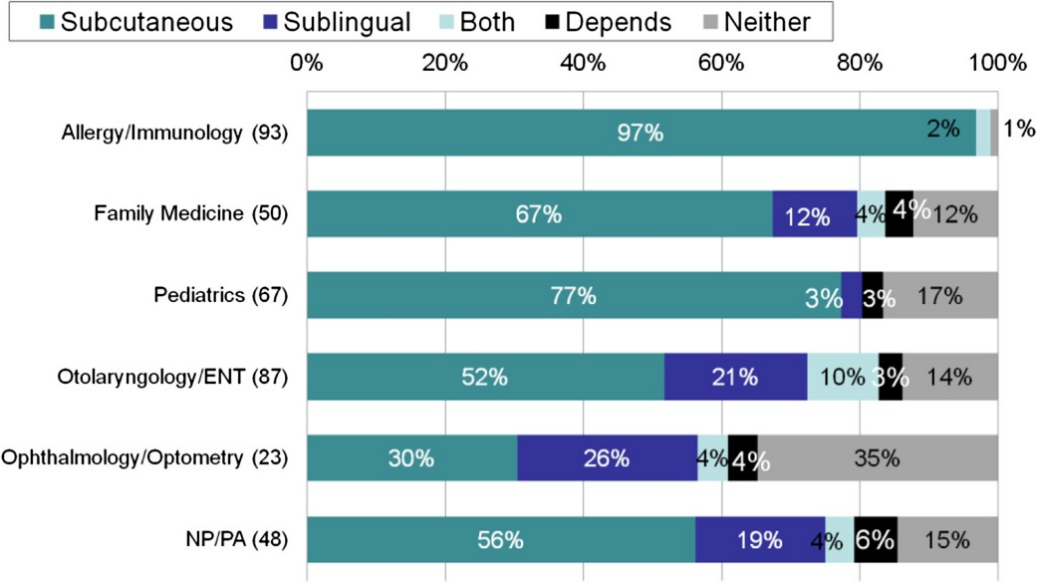
^**4**^**Providers’ immunotherapy prescribing practices.** Prescribing practices of subcutaneous and sublingual immunotherapy as a function of provider’s specialty. NP/PA (Nurse Practitioners/Physician Assistants).

The Allergies, Immunotherapy & Rhinoconjunctivitis Survey (AIRS) [3] surveyed both patients and health care providers during a 2-month period in 2012. Patients were eligible if they were 5 years of age or older, had a diagnosis of hay fever, allergic rhinitis, rhinoconjunctivitis, or nasal or eye allergies, and either had symptoms or medication use in the prior 12 months. Providers were eligible if they provided direct patient care to patients with allergies in an outpatient setting at least weekly. 2765 patients and 500 providers completed the survey. Patients were taken from a national probability sample screened for a geographic stratification sampling over 43,000 households in the US. Providers were multidisciplinary, including practitioners of allergy, family medicine, otolaryngology, ophthalmology/optometry, and pediatrics. In addition to physicians, nurse practitioners and physician assistants were surveyed as well. The primary reason that practitioners across the board recommended immunotherapy to both children and adults was lack of efficacy of other therapies. When asked if the immunotherapy recommended was subcutaneous or sublingual (Figure 1), responses varied greatly by specialty. 97% of allergists recommended subcutaneous, whereas 2% recommended both, and 1% recommended neither [4]. All practitioners recommended subcutaneous more often than sublingual, though ophthalmologists (26%) and otolaryngologists (21%) were more likely to recommend SLIT than other providers.

### Difficulty getting FDA approval

FDA approval has been difficult because SLIT studies differ from allergy and asthma medication studies in terms of population selection, symptom variance, and exposure variance. In all medication studies, there is population variation to account for, but SLIT studies must account for variation in allergen exposure as well. Unlike studies for allergic rhinitis medications, patients are not symptomatic prior to treatment. Furthermore, pollen levels vary, which will affect the degree of symptoms seen during a study.

Total composite scores (TCS) to assess improvement from SLIT for allergic rhinitis and rhinoconjunctivitis rely on symptom improvement and decreased medication use to prove effectiveness. Symptom scores are generally based on standardized scoring symptoms evaluating nasal and ocular symptoms. Scores to evaluate medication use, however, vary by study without standardized norms to make effective comparisons across studies. This lack of standardized composite score makes interpretation of SLIT studies more complicated.

Medication studies generally require a statistically significant change in the study group from control (p < 0.05) to prove efficacy, but, according to the FDA, that is not enough to prove a true clinical meaningful change in SLIT studies. The FDA requires that studies demonstrate 10% efficacy above the 95% confidence interval in mean TCS difference versus placebo and at least a 15% improvement in TCS compared to placebo. Other organizations have suggested at least a 20% improvement in TCS versus placebo, as recommended by the World Allergy Organization [5].

### Sublingual immunotherapy products

Currently, the extracts being used by practitioners for sublingual immunotherapy are commercially available extracts used for SCIT in the US. SLIT tablets for northern pasture grasses and ragweed are approved by the FDA for use In the US. Results of clinical trials addressing safety and efficacy of these products, as well as of a ragweed sublingual liquid currently pending FDA approval, are summarized in Table 1.

**Table 1:** **Summary of US SLIT trial findings**

Study	Product Studied	Number of patients in study	Age in years, mean (range)	% Poly-sensitized	Primary Efficacy Endpoint Results	Adverse Events Summary
Nelson et al. 2010	Timothy grass tablet	439	35.9 (18–65)	76.9%	• Compared to placebo, the treatment group had at 20% improvement in TCS.	• The most common AEs were oral pruritus, throat irritation, ear pruritus, and upper respiratory tract infections.
					• DSS improved by 18% and RQLQ by 17% in treatment group versus placebo. DMS improved by 26%.	• There were no serious AEs or anaphylactic reactions.
						• There was 1 patient in the treatment group that received epinephrine from possible systemic reaction.
Blaiss et al. 2010	Timothy grass tablet	345	12.3 (5–17)	89%	• TCS improved 26% in treatment group versus placebo.	• Common AEs included oral pruritus, throat irritation, and mouth irritation.
					• DSS improved 25%, DMS improved 81%, and RQLQ improved 18% over placebo.	• A moderate reaction of lip edema, dysphagia, and cough was reported by one patient, which responded to epinephrine. It was not considered systemic by the investigator.
Maloney et al. 2014	Timothy grass tablet MK-7243	1501	33 (5–65)	85%	• MK-7243 provided a 20% lower DSS during peak and entire GPS, 38% lower DMS during peak season, 35% lower DMS during entire GPS, and 32% lower TCS during entire GPS compared to placebo.	• The most common AEs were throat irritation, oral pruritus or paresthesias, mouth edema, and ear pruritus.
						• Systemic reactions occurred in 2 MK-7243 patients, were moderate in severity, and resolved without treatment.
						• There were no severe systemic reactions and no deaths.
Cox et al. 2012	5-grass tablet 300IR	473	37.2 (18–65)	77.9%	• Those in the treatment group had a significantly lower CS during the peak pollen season than the placebo group, with a relative decrease in 28.2%.	• Common AEs included oral itching, throat irritation, and nasopharyngitis.
					• The benefit was most notable in those with baseline detectable serum IgE to timothy grass.	• There were no anaphylactic reactions and no serious treatment-related adverse events.
Nolte et al. 2013	Ragweed tablet	565	35.4 (18–50)	85%	• During the peak ragweed season, the 6- Amb a 1 unit group and 12-Amb a 1 unit group had a 21% and 27% reduction in TCS compared to placebo, respectively.	• The most common AEs were oral pruritus, throat irritation, swollen tongue, and ear pruritus.
					• During the entire ragweed season, the 6- Amb a 1 unit group and 12-Amb a 1 unit group had a 16% and 26% reduction in TCS compared to placebo, respectively.	• There were no serious treatment-related AEs.
						• One treatment patient received epinephrine for sensation of pharyngeal edema.
Creticos et al. 2013	Ragweed liquid	429	38.3 (18–55)	83.9%	• The treatment group had a 43% reduction in TCS during the entire ragweed season compared to placebo.	• The most frequently reported treatment-related AEs were oromucosal reactions, which occurred early and were transient.
					• This was statistically significant after adjusting the 95% confidence interval to account for 20% clinically meaningful difference over placebo.	• There were no systemic reactions, anaphylaxis, or death in the treatment group.

*TCS* Total Combined Score, *DSS* Daily Symptom Score, *DMS* Daily Medication Score, *RQLQ* Rhinoconjunctivitis Quality of Life Questionnaire, *AEs* Adverse events, *GPS* Grass pollen season, *CS* Combined Score.

**Figure 2:**
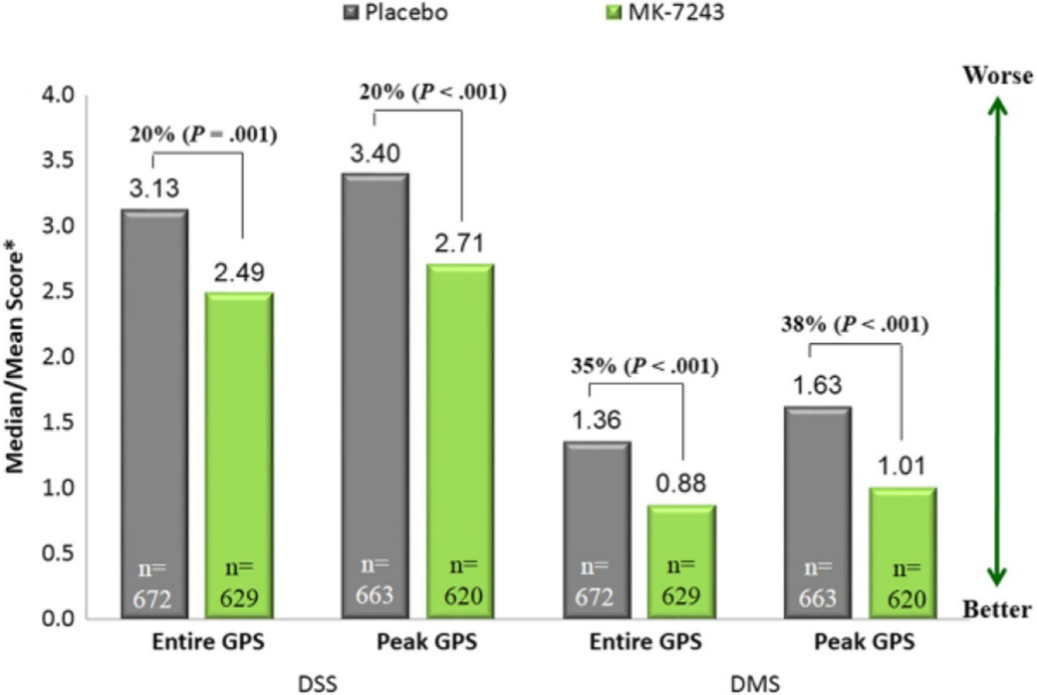
^**6**^**Daily symptom and medication scores with timothy grass sublingual tablet.** Daily symptom score (DSS) and daily medication score (DMS) over the entire and peak grass pollen season (GPS). *Medians are reported for DSS, and means are reported for DMS. Between-treatment differences in DSS for MK-7243 vs placebo were -0.64 over the entire GPS and -0.69 over the peak GPS. Between-treatment differences in DMS for MK-7243 vs placebo were -0.48 over the entire GPS and -0.62 over the peak GPS.

Several clinical trials have evaluated the safety and efficacy of timothy grass SLIT tablets. The largest clinical trial to date [6] evaluated the safety when only the first dose of timothy grass tablet immunotherapy was given in the physician’s office. Prior North American studies in both adult and pediatric populations [7, 8] evaluated safety when the first three doses were administered in the physician’s office, without evidence of an additional safety advantage. Subjects for the most recent clinical trial were 5 to 65 years of age, with history of grass pollen-induced allergic rhinitis (AR) or allergic rhinoconjunctivitis (ARC), with or without asthma [6]. Subjects must have been treated for AR/C during the previous grass pollen season (GPS), have a positive skin prick test to *Phleum pratense* (≥5 mm wheal) and/or specific IgE against *Phleum pratense* (≥0.7 kU/L). FEV_1_ had to be ≥ 70% of predicted value. There were no significant differences in baseline demographic characteristics between the placebo and treatment groups. The majority (85%) of each group was polysensitized to other aeroallergens besides grass. Total combined scores (TCS) were determined during peak GPS and 16 weeks prior, using combination of Daily Symptom Score (DSS) and Daily Medication Score (DMS), which factored in use of oral antihistamines, ophthalmic antihistamines, intranasal steroids, and oral steroids. Those receiving timothy grass tablets had 20% lower DSS than placebo during both the peak GPS and entire GPS (Figure 2). They also had 38% and 35% lower DMS during peak and entire GPS, respectively. The TCS for the entire season was 23% lower in the treatment group. These were similar to rates found in prior studies by Nelson et al. [7] and Blaiss et al. [8] When examined exclusively in children age 5–17 years, the TCS was 32% lower in the treatment group during the entire GPS. The most commonly reported adverse event in both the grass AIT and placebo groups was throat irritation (24% and 4% of subjects, respectively). Other common adverse events were oral pruritus, oral paraesthesia, mouth edema, and ear pruritus. Systemic allergic reactions were rare in the studies.

**Figure 3:**
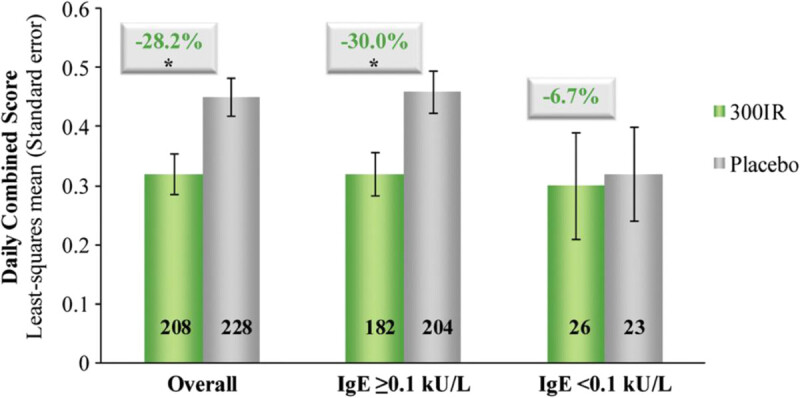
^**9**^**Daily combined scores with 300 IR 5-grass sublingual tablet as a function of specific IgE.** Daily CS overall and in sub-groups based on timothy grass-specifc serum IgE at baseline. The number of participants in each group is displayed in each bar. Note: IgE data were not obtained for 1 placebo-treated subject. *P < .001 versus placebo.

A study addressing the clinical efficacy and safety of a 300 index of reactivity (IR) 5-grass pollen sublingual tablet was performed in adults in the United States [9]. 473 subjects with documented grass pollen allergy by history and positive skin test and Rhinoconjunctivitis Total Symptom Score of 12 or greater (scale 0–18) during the prior grass pollen season were randomized in a double-blind, placebo-controlled trial to receive 300IR 5-grass sublingual tablet or placebo starting 4 months prior to the pollen season and continuing through the season. Efficacy was determined using a combined score (CS) that took into account both daily symptoms and medication use. The treatment group had significantly lower CS scores during the peak pollen season than the placebo-treated group. Patients also had serum specific IgE and IgG_4_ to timothy grass measured at baseline, at the midpoint of the pollen season, and at the end of the season. Patients without measurable baseline specific IgE to timothy grass showed no difference between placebo and active treatment. In patients with measurable serum specific IgE to timothy grass, those treated with 300IR had an increase in IgE over the course of the pollen season, while those treated with placebo remained relatively unchanged. Similarly, timothy grass serum IgG_4_ increased in the treatment group but remained unchanged in the placebo group. Figure 3 shows daily CS as a function of serum specific IgE to timothy grass, demonstrating that patients with undetectable IgE had little difference between treatment and placebo groups. This indicates that detection of serum specific IgE to timothy grass, and not just a positive allergy skin test, may be an important determinant in deciding who is a good candidate for treatment, though per FDA labeling the only requirement is either a positive skin test or blood study for all SLIT products. Frequently reported adverse events were mild, including oral itching, throat irritation, and nasopharyngitis. There were no episodes of anaphylaxis, and no treatment-related serious adverse events.

**Figure 4:**
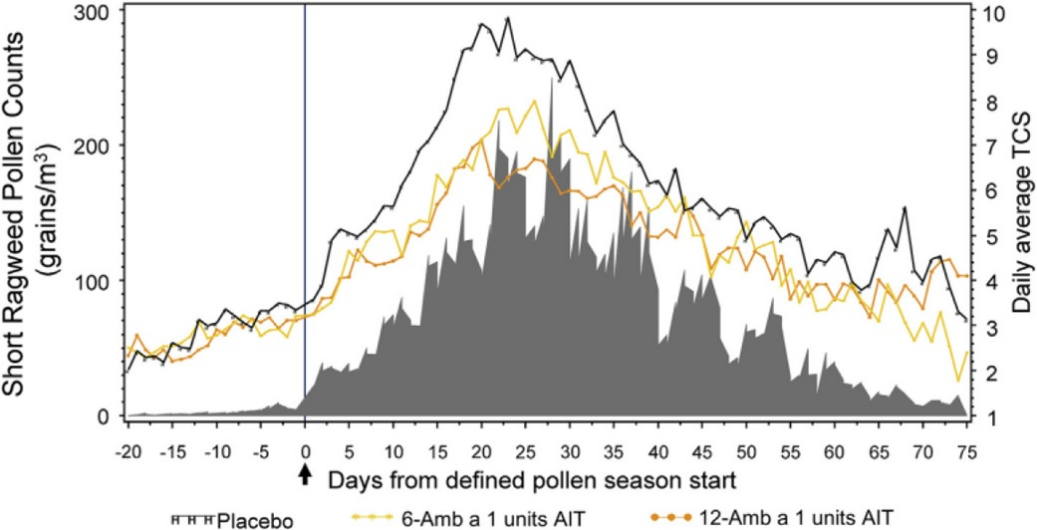
^**10**^**Total combined scores for ragweed sublingual tablets versus placebo.** Total combined score (TCS) plotted against pollen count. AIT indicates allergy immunotherapy tablet.

A study by Nolte et al. evaluating a sublingual ragweed immunotherapy tablet [10] showed that subjects treated with 6 units of Amb a 1 and 12 units of Amb a 1 both showed decrease in TCS compared to placebo. Though there was no significant difference between the two, a more pronounced clinical efficacy was noted with the 12 Amb a 1 unit dose (Figure 4). Similarly, both showed decrease compared to placebo in oral antihistamine, ocular antihistamine, and nasal corticosteroid use with a slightly larger decrease in medication use in the 12 units of Amb a 1 group compared to the 6 unit group. Changes in TCS during peak and entire pollen season were similar. In patients rating symptoms using a visual analogue scale, there was significantly better improvement with the 12 versus 6 Amb 1 unit dose compared to placebo. Adverse events were mild and generally involved oropharyngeal itching or discomfort. One patient did receive epinephrine for sensation of pharyngeal edema.

Creticos et al. [11] performed a phase 3, randomized double-blind, placebo-controlled parallel trial of standardized short ragweed (RW) sublingual allergy immunotherapy liquid (SAIL) for allergic rhinoconjunctivitis in North America. Subjects, aged 18–55 years, with or without asthma, were selected based on symptom severity (at least a 2 year history of moderate to severe rhinoconjunctivitis secondary to ragweed) and positive skin prick test to short ragweed. Subjects self-administered RW-SAIL or placebo (in a 1:1 ratio) starting 8–16 weeks prior to the 2011 RW season, and continued through the season. The SAIL group had incremental increase in doses at beginning of the study, starting with placebo, 18 micrograms Amb a 1, and then 50 micrograms Amb a 1, and continued the highest tolerated dose through the rest of the season. Subjects maintained daily symptom and rescue medication e-diaries. Efficacy endpoints included total combined symptom + medication scores (TCS), daily symptom scores (DSS), IgG4 and IgE ragweed-specific antibody. Safety was evaluated by adverse event diaries, laboratory evaluation, and physical examinations. During the entire season, there was a 43% decrease in TCS in the RW-SAIL group compared to placebo. Similarly, there was a 42% decrease in TCS during peak season, and decrease in DSS during entire (42%) and peak (41%) seasons in the RW-SAIL group compared to placebo. 94% of patients in the treatment group tolerated the maximum dose (50 micrograms of Amb a 1), with 6% remaining on, or stepping down to, the lower dose of 18 micrograms. There were no systemic reactions, anaphylaxis, or deaths in the RW-SAIL group. The most common treatment emergent adverse effects (AEs) were headache, upper respiratory symptoms, and gastrointestinal symptoms. The most frequent treatment-related AEs were oromucosal reactions, which occurred early and were transient. There were no significant findings on laboratory monitoring, aside from one patient with hematuria that was deemed not related to the treatment. One patient in the RW-SAIL group had angioedema of the hand after touching a household cleaner, that was treated with epinephrine in the emergency department. It was judged not likely related to the study drug.

### Other concerns

There has been considerable focus on safety of SLIT versus SCIT and what this means for the patient and clinicians alike. One of the potential benefits of SLIT is that it is home-based therapy due to the excellent safety profile, which may increase adherence and convenience for patients. There have no deaths reported with SLIT home use throughout the world. Clinical trials in the US, as discussed, have found systemic reactions to be rare. When systemic reactions do occur, they are generally mild or moderate in nature, and not life-threatening. In fact, most reactions resolve without treatment. Due to this, there is ongoing debate over whether patients on SLIT would require epinephrine auto-injectors. Table 2 shows the pros and cons of patients having an epinephrine auto-injector for SLIT.

**Table 2:** **Pros and cons for prescribing auto-injectable epinephrine for SLIT**

Pros	Cons
● There is a slight, though rare risk for anaphylaxis.	● Adverse reactions with SLIT are generally mild, and systemic reactions are generally not severe, with no deaths reported.
● SLIT is a home-based therapy without anaphylaxis-trained practitioners available should a reaction occur	● Auto-injectable epinephrine may be used incorrectly or inappropriately by patients.
● There are medical-legal concerns if a patient should have a reaction at home without having an auto-injector prescribed.	● Auto-injectors are not required for SLIT in Europe and Canada. Many US practitioners do not require them for SCIT patients.
	● Costs of epinephrine autoinjectors

Dosing of the approved SLIT tablets is different than what US clinicians are used to doing with SCIT. For the 5-grass tablet, the FDA label states to start 16 weeks before the start of the grass season and continue through the season, while the timothy grass tablet and ragweed tablet begin12 weeks before the start of the appropriate season and continue through that season. Also, the timothy grass tablet has a second dosing schedule year- round for 3 years for sustained effectiveness.

There are multiple cost concerns for both the patient and clinician as well. FDA-approved SLIT will be a prescription benefit, and coverage may be dramatically different than coverage for SCIT, which is a procedure prepared and billed for by an allergist. Cost of therapy, whether through insurance or out-of-pocket, will likely contribute significantly to adherence as well. González-De-Olano et al. [12] looked at adherence prior to and during a recent Spanish recession, and showed a significant decrease in both SCIT and SLIT use during the recession. Ongoing changes in the healthcare system in the United States may cause changes in IT coverage across the board, whether SLIT or SCIT. This may provide new challenges when making the choice between therapies, and will have to be taken into consideration as these changes become evident.

Lastly, adherence to therapy and persistence with therapy is a concern for home-based therapy as well. Patients receiving SCIT have observed therapy, and practitioners know when patients are not receiving their shots. Adherence to home-based SLIT is more difficult to monitor. A retrospective chart review of patients in a US allergy practice utilizing both SCIT and SLIT showed that only 32.5% of all patients completed the entire treatment course defined as 3 years, 35% of SCIT patients and 23.7% of SLIT patients [13]. SCIT patients remained on therapy for an average of 3.6 years, compared to 2.6 years for SLIT patients.

## Conclusions

Several double-blind, placebo-controlled, randomized control trials have been performed in North American for the different forms of SLIT being reviewed for approval by the FDA. These studies have demonstrated clinical effectiveness in terms of symptom and medication scores, with low incidence of adverse effects. In general, adverse effects of SLIT have been mild, with the most common adverse effects being oromucosal itching, swelling, or pain.

Assuming SLIT treatments are approved in the US, there are numerous challenges on the horizon for allergists and other practitioners. It is unclear if clinicians will use approved SLIT therapies, continue to mix their own SLIT from SCIT extracts, or only use SCIT. SLIT is a promising option for monosensitized and some polysensitized patients, those that refuse injections, especially children, and patients that can not come regularly to a clinician’s office for injections. SCIT may continue to be a superior option for some polysensitized patients, particularly when sensitized to aeroallergens for which approved SLIT treatments are not available. Monitoring of adherence to therapy would be more difficult with SLIT compared to SCIT, but a home-based therapy not requiring weekly office visits may actually improve patient adherence.

There are financial aspects to consider as well. SCIT is a billable procedure, whereas SLIT would be a prescription. Changes in the current healthcare system in the United States may affect coverage of both SCIT and SLIT. Depending on the patient’s insurance coverage, one modality of immunotherapy may be more financially favorable to the patient, which will likely lead to its use for that patient. Lastly, allergists may face a change in income should SLIT become common practice, with the loss of profit from SCIT procedure billing. However, SLIT may grow the allergist’s practice, as more patients may be receptive to a home immunotherapy program. All of these concerns will be have to taken into consideration as FDA-approved SLIT therapies become available.

## Authors’ original submitted files for images

Below are the links to the authors’ original submitted files for images. Authors’ original file for figure 1 Authors’ original file for figure 2 Authors’ original file for figure 3 Authors’ original file for figure 4

## Abbreviations

SLIT: Sublingual immunotherapy
ACAAI: American College of allergy, asthma, and immunology
FDA: Food and drug administration
RW: Ragweed
SAIL: Sublingual allergy immunotherapy liquid
TCS: Total combined symptom + medication score
DSS: Daily symptom scores
CS: Combined score
LS: Least squares
AEs: Adverse events
AR: Allergic rhinitis
ARC: Allergic rhinoconjunctivitis
GPS: Grass pollen season
FEV_1_: Forced expiratory volume in 1 second
DMS: Daily medication score
IR: Index of reactivity
RQLQ: Rhinoconjunctivitis quality of life questionnaire.
