# Supplementary figures and images for: Current status of sublingual immunotherapy in the United States
Source: World Allergy Organ J. 2014 Oct 8;7(1):24. doi: 10.1186/1939-4551-7-24 (PMC4194410; doi:10.1186/1939-4551-7-24)

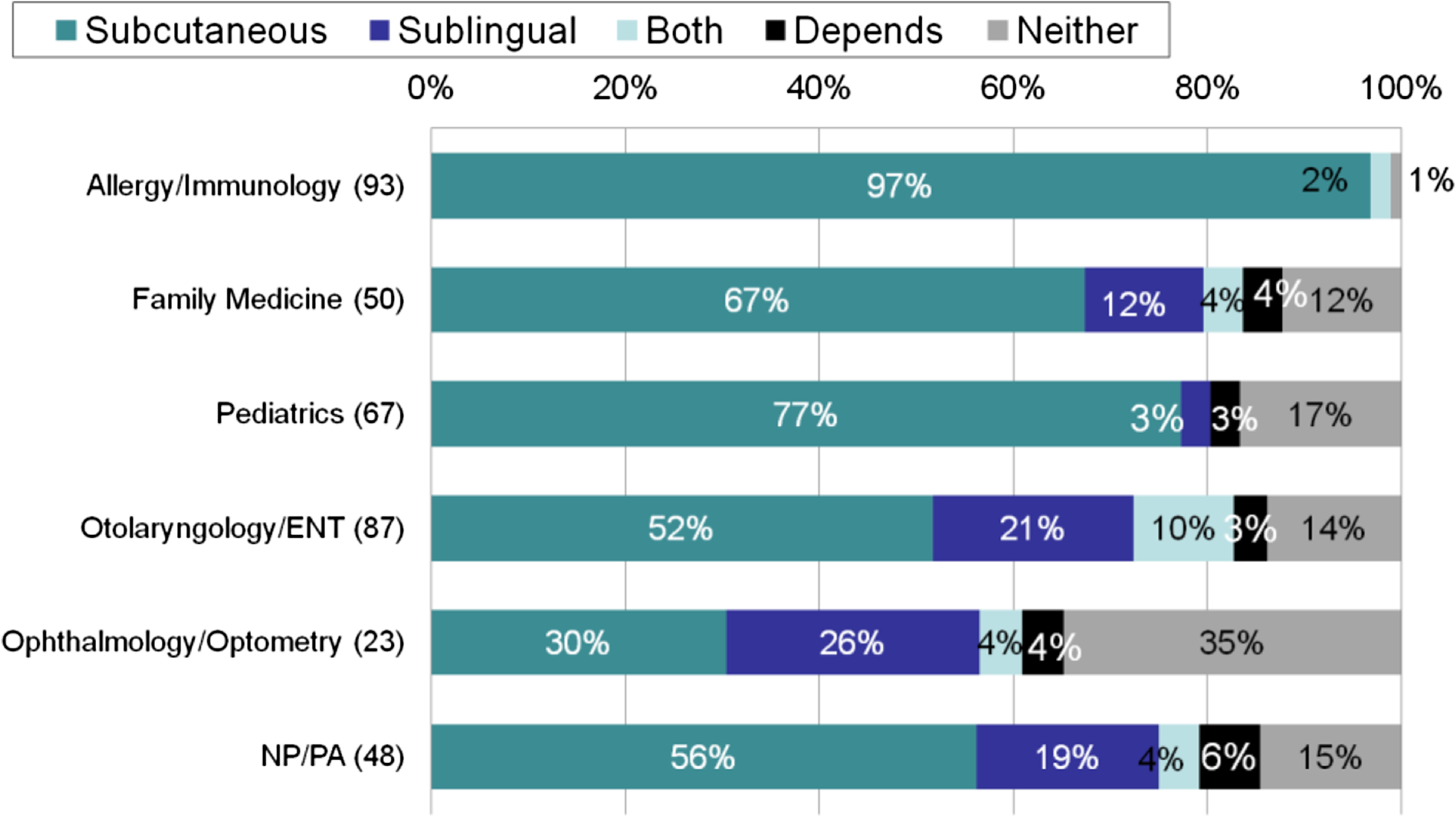

Supplement: Supplementary file 1 — Authors’ original file for figure 1 [file 40413_2014_67_MOESM1_ESM.tiff]

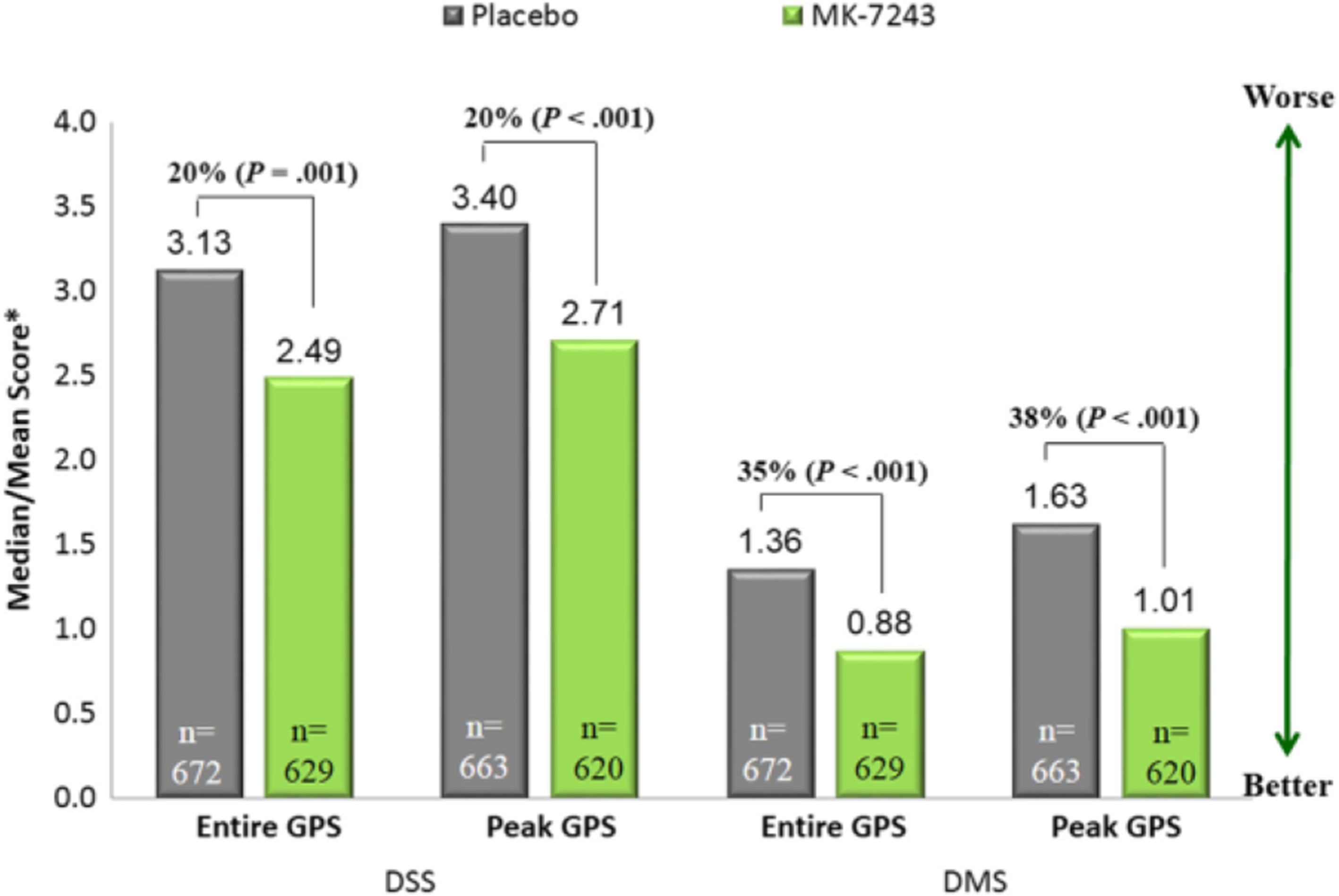

Supplement: Supplementary file 2 — Authors’ original file for figure 2 [file 40413_2014_67_MOESM2_ESM.tiff]

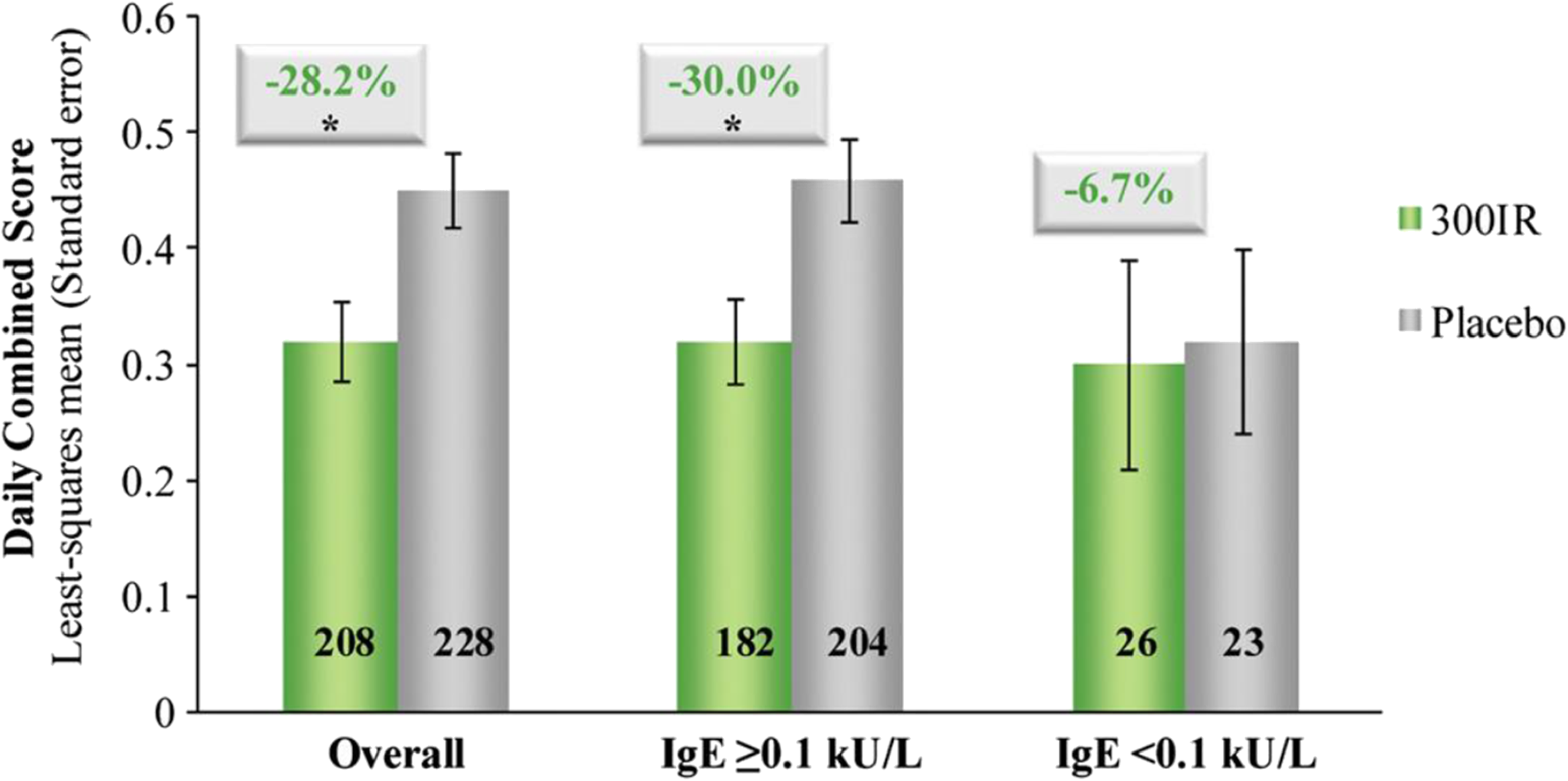

Supplement: Supplementary file 3 — Authors’ original file for figure 3 [file 40413_2014_67_MOESM3_ESM.tiff]

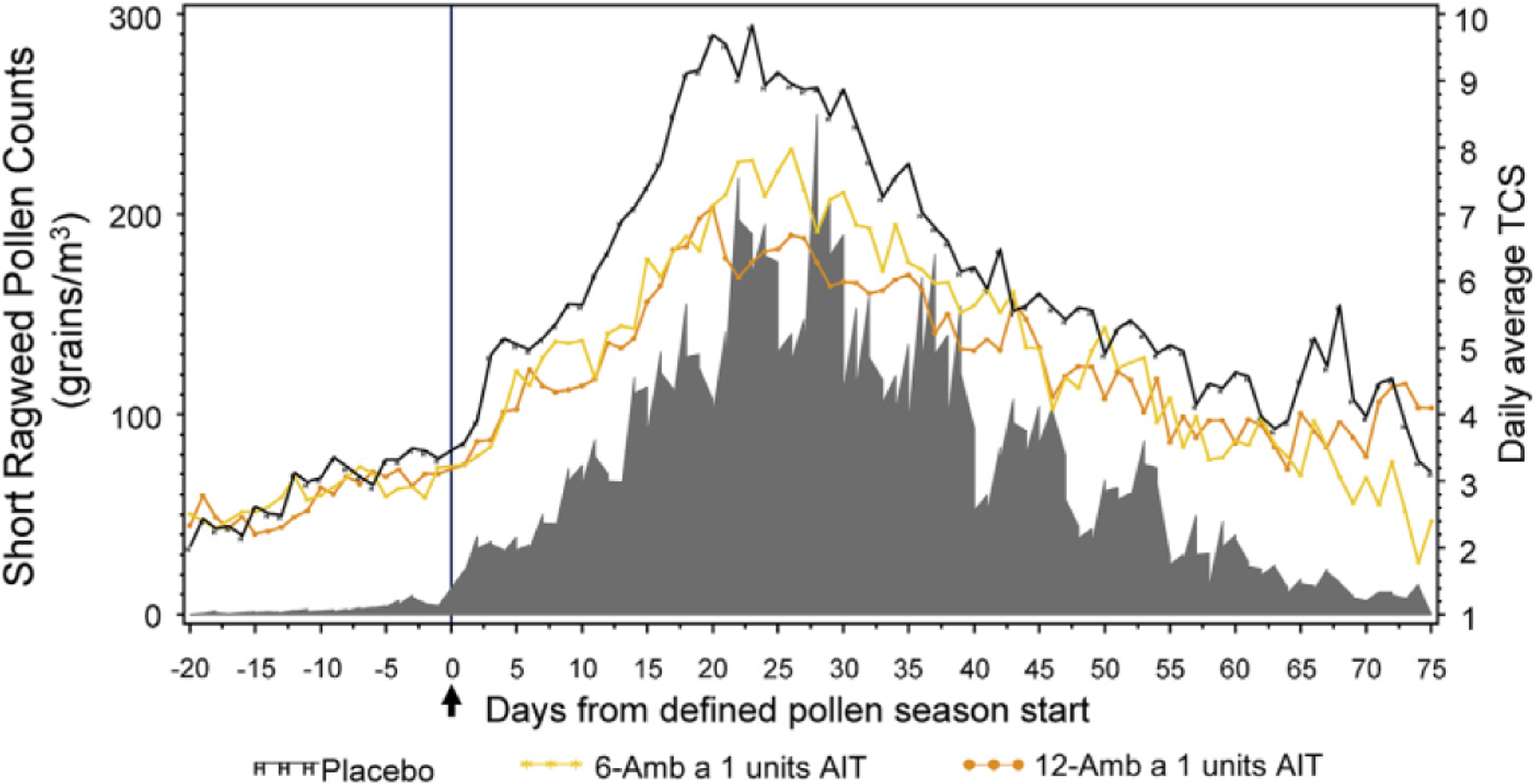

Supplement: Supplementary file 4 — Authors’ original file for figure 4 [file 40413_2014_67_MOESM4_ESM.tiff]
